# Attitudes toward Telling the Truth to Cancer Patients in Iran: A Review Article

**Published:** 2017-07-01

**Authors:** Ali Karimi Rozveh, Reza Nabi Amjad, Javad Karimi Rozveh, Davood Rasouli

**Affiliations:** 1 School of Nursing and Midwifery, Tehran University of Medical Sciences, Tehran, Iran; 2 School of Nursing and Midwifery, Alborz University of Medical Sciences, Karaj, Iran; 3 School of Medicine, Alborz University of Medical Sciences, Karaj, Iran; 4 School of Medical Education Sciences, Shahid Beheshti University of Medical Sciences, Tehran, Iran

**Keywords:** Telling the truth, Cancer patient, Breaking bad news, Review article, Iran

## Abstract

**Background:** *Patients* generally *have the right to be informed* of their condition, but the debate over the issue of truth disclosure is still present. The attempt of this study is to review the approaches toward truth- telling to cancer patients in Iran.

**Materials and Methods: **This study is a narrative review that included articles published in Iran on attitudes toward telling the truth to cancer patients. The present study extracted data from articles published in PubMed, Science Direct, Scientific Information Database (SID), Magiran, Iran Medex, Google Scholar, Iranian Research Institute for Information Science and Technology with key terms such as truth disclosure, breaking bad news, death awareness and disclosure of diagnosis without any time restriction.

**Results: **Totally, 21 articles including 14 in English and 7 in Persian were selected and reviewed**. **The results of the study have shown that although treatment team and caregivers are unwilling to disclose the truth to patients, they have a tendency to obtain more information about their disease.

**Conclusion: **As the incidence of cancer has increased worldwide, telling the truth to patients seeking more information about cancer disease would be inevitable, but more studies are required to provide scientific procedures based on evidence for truth disclosure, not the whole, to cancer patients.

## Introduction

 According to Medical Ethics, patients have the right to know their medical status^1^. Despite patients’ rights about knowing the truth, concealment of diagnosis is a great problem in health care system^2^. Studies have shown that there is an association between patients' information about prognosis of their disease and their treatment preferences^2^^,^^3^. All patients with cancer should be told the truth about disease to help them make their own decision about treatment choices. It has been shown that this approach may positively affect patient satisfaction^4^.

There are cultural, professional and emotional obstacles to revealing the truth to cancer patients^5^^, ^^6^^.^ In Iran, patients with cancer do not have enough information about their disease and treatment choices. Meanwhile, they are not satisfied with the information they receive from their health care providers and have a tendency to obtain more information^7^. It has been shown that a good *doctor–patient *relationship does not exist and a lack of experience in delivering bad news may contribute to negative emotional reactions and loss of hope in patients^8^.Cultural differences in telling the truth to cancer patients have generated controversy among caregivers and patients^3^^,^^9^.Findings from one culture cannot be applied to other cultures for making decisions, so this study reviewed the previous studies conducted in Iran to find appropriate answers to the following questions:

- What are the attitudes of physicians and nurses toward telling the truth to cancer patients?
- Do the cancer patients and their families have tendency in facing reality?
- What are the consequences of making the patient aware of cancer diagnosis on the treatment procedure?

## MATERIALS AND METHODS

 This study is a narrative review including published articles (in English and Persian) in Iran on attitudes toward telling the truth to cancer patients. The present study extracted data from articles published in PubMed, Science Direct, Scientific Information Database (SID), Magiran, Iran Medex, Google Scholar, Iranian Research Institute for Information Science and Technology with key terms such as truth disclosure, breaking bad news, death awareness and disclosure of diagnosis without any time restriction. In total*, *50 related articles were found; of which 19 were on psychological aspects of cancer. Four articles were excluded from the study due to same content. Articles were available in full-text format. The type of study, the time of study and the sample size are shown in Table 1.

**Table 1:** studies related to truth-telling in Iran

**Authors **	**Title**	**Type of study**	**Sample size**
Ghazanfari et al. 2014	Nurses and physicians attitude toward truth-telling to cancer patients	Descriptive	22 physicians, 32 nurses
Valizadeh et al. 2014	Truth-telling and Hematopoietic Stem Cell Transplantation: Iranian Nurses’ Experiences	Qualitative	18 nurses
Seyedrasooly et al.2014	Association between Perception of Prognosis and Spiritual Well-being among Cancer Patients	Descriptive	200 patients
Motlagh et al.2014	Attitude of Cancer Patients toward Diagnosis Disclosure and their Preference for Clinical Decision-making: A National Survey	Descriptive	1226 patients
Rezaei et al.2014	Identifying Appropriate Methods of Diagnosis Disclosure and Physician- Patient Communication Pattern among Cancer Patients in Iranian Society	Qualitative	40 patients
Arbabi et al. 2014	Patients’ Preference to Hear Cancer Diagnosis	Descriptive	200 patients
Joibari et al.2013	Nursing Experience of Breaking Bad News to Patients Caregivers	Qualitative	30 nurses
Izadi et al.2013	Nursing Experience of Telling the Truth: A Phenomenological Study	Qualitative	14 nurses
Valizadeh et al. 2012	Cancer Disclosure: Experiences of Iranian Cancer Patients	Qualitative	20 patients
Lashkarizadeh et al.2012	Cancer Patients Attitudes toward Disclosure of Diagnosis and Notification	Descriptive	385 patients
Zamani et al. 2011	Physicians and Patients Attitudes toward How truth-telling to Cancer Patients	Descriptive	50 physicians & 150 patients
Beyraghi et al. 2011	Disclosure of Cancer Information in Iran: a Perspectiveof Patients, Family Members and Health Professionals	Qualitative	8 patients& 5 caregivers& 6 nurses & 7 physicians
Faridhosseini et al. 2010	Disclosure of Cancer Diagnosis: what Iranian Patients Do Prefer?	Descriptive	126 patients
Kazemi et al.2010	Assessment of Aspects of Telling the Truth from View of Medical Team of Tabriz University of Medical Sciences	Descriptive	200 physicians
Bagherian et al.2010	Attitude of Nurses who Working in Valiasr Hospital and Cancer Institute toward Caring of Dying Patients	Descriptive	120 nurses
Montazeri et al. 2009	Disclosure of Cancer Diagnosis and Quality of Life in Cancer Patients: Should It Be the Same Everywhere?	Descriptive	142 patients
Tavakoli et al, 2008	Educating Doctors About Breaking Bad News: An Iranian Perspective	Qualitative	10 physicians
Shahidi et al. 2007	Truth-telling to Cancer Patients from Relatives Point of View: A Multi- centre Study in Iran	Descriptive	155 caregivers
Larizadeh et al. 2007	Assessment of Cancer Patients Awareness About Own Disease	Descriptive	150 patients
Tarighat et al.2006	Assessment of Knowledge and Satisfaction in Cancer Patients of Received Information About Own Disease and Its Relation to Anxiety and Depression in Institute Caner of Imam Khomeini Hospital, 2014-2015.	Descriptive	250 patients
Montazeri et al.2004	Does Knowledge of Cancer Diagnosis Affect Quality of Life? AMethodological Challenge	Descriptive	129 patients

## Results

 The survey included 50 articles published in English and Persian on telling the truth to patients with cancer. Totally, 21 articles including 14 in English and 7 in Persian were selected and reviewed; of which 7 were qualitative and 14 were descriptive. The study included physicians (n=767), nurses (n=226), patients (n=3470) and patients' caregivers (n=160).The sample size varied between 10 and 230 participants. Twenty-nine articles were omitted because of duplication and low quality. Data analysis revealed 3 categories: awareness and attitude of treatment team towards truth disclosure, awareness and attitude of patients and their families towards truth disclosure and the effects of truth telling on patients.

## Discussion

**Awareness and attitude of treatment team towards truth disclosure**

There are two attitudes toward truth disclosure: some physicians presume that cancer patients ought to be informed of *their* illness, but others conceal the diagnosis from patients. Contrary to East Asian countries, patients are *informed of their cancer* diagnosis in most western countries^10^. The issue of truth-telling regarding cancer diagnosis is still largely unresolved^7^. Multiple surveys have been conducted on disclosure of information to cancer patients in Iran. In a study conducted by Ghazanfari et al., 85.2% of participants consisted of physicians and nurses (n=51) believed that a patient with cancer should be told the truth, but it is the physician who makes the final decision^11^. According to Izadi et al. (2013), nurses believed in telling patients the truth to increase patient confidence and satisfaction, decrease the number of law suits against nurses and physicians, make informed decisions, maintain and strengthen the relationship between patient and nurse ,fulfill Islamic religious obligations, and obtain patient’s cooperation in treatment procedure^12^.In another study, 90% of physicians stated that the cancer patients should be told the truth in the early stages ,while 72 % believed patients should be told in advanced stages of the disease^13^. Kazemi et al. (2010) showed 20% of physicians agreed that cancer patients should be informed of their diagnosis and the terminal nature of their illness^14^. Most of 400 physicians surveyed in one study preferred to disclose the full truth to their patients and 78% believed that providing patients with information would facilitate the treatment procedure and *decrease the*number of malpractice lawsuits^14^. In contrast, Valizadeh et al. (2014) found that nurses preferred to withhold the truth from patients^15^. They believed that truth disclosure would create tensions, negative emotional reaction and destroy the notion of secrecy^12^.

In a survey conducted by Beiraghi et al. (2011), study participants (nurses and physicians) believed that disclosure of cancer diagnosis would be a mistake^16^. Another study on 30 nurses has shown that they use indirect approach to break bad news based on the physician's advice^17^. In a study by Bagherian et al. (2008) on nurses' attitudes (n=120) toward truth telling, the authors found that they were neither willing nor able to train or talk about dying^18^.

The majority of physicians (n=20) interviewed in another survey preferred to inform close family members rather than the patients directly. They stressed that patients should be told the truth about their diagnosis by the physician in the vague or indirect language^19^. Another study found that physicians used the patriarchal model or religious beliefs to disclose the bad news. Developing an integrated curriculum with special emphasis on disclosure of bad news and communication skills seem to be essential for physicians^20^. 

**Attitudes of patients and family members toward truth-telling**

Being informed of cancer diagnosis is one of the factors affecting the quality of life cancer patients^10^. Some surveys show that cancer patients should be informed about the nature of the disease to make informed decision, therefore, when the patients are not told the truth they can no longer make informed choices^21^. Sometimes, physicians are asked to withhold the truth from patients to avoid taking away hope or causing them severe distress^10^.

A survey conducted by Motlagh et al., (2014) indicated that the majority of cancer patients wanted to be informed of the diagnosis of cancer or prognosis and preferred some form of shared or collaborative decision making with the physicians, but only 46.7% (n=565) of study participants were informed of the diagnosis^22^. Another study of 200 patients showed that only 73% were informed of their true diagnosis and the majority of whom (93%) wanted the disclosure of diagnostic information. Meanwhile, 75.5% preferred to be informed only by the physician^23^. Rezaei et al’s. (2014) study indicated that 80% of patients wanted to be informed of the diagnosis of cancer after confirmation. The study also revealed that patients wanted to be told the truth by the physician^24^.

In another study of patients with cancer (n=385), all study participants wanted to be informed of the prognosis and side effects of their disease. In response to the question” who should be informed first?”, 87% stated patients should be told the truth by the first physician who made the diagnosis. The only statistically significant variable among demographic data of patients was sex. Male patients were more interested in being informed of their disease than females. However, most of patients (60%) were not informed of their diagnosis^10^.

The results of the study by Zamani et al. (2011) on 150 cancer patients towards truth telling indicated that 88% agreed that the patients should be told the truth in the early stages, and 78% agreed that truth should be told to patients in the terminal stages of cancer^13^.

A study by Farid Hosseini et al., (2010) on 126 patients with cancer showed that 60.31 % were informed of their diagnosis, and 88% who were completely unaware of their diagnosis preferred to be directly informed of their disease by the physician^25^. 

Another survey on patients' awareness of their condition showed that 53 (35.3%) of 150 patients were informed of their cancer diagnosis. Fifty-eight patients(38.7%) were involved in the selection of their treatment plan. The patient’s awareness of the diagnosis was related to education, treatment type and place of health care service delivery. Meanwhile, patients who underwent radiation therapy had higher levels of education and awareness. In the current study, 35.3% of patients were aware of the diagnosis of cancer, while only 7.3% were aware of prognosis. Furthermore, 58 patients (38.7%) were involved in the different stages of treatment; of whom 88.7% Vs.11.3% knowingly engaged in the treatment plan .The results of the study showed that there was a close relationship between the level of awareness of patients and their involvement in the therapy program^10^.

Tarighat et al. (2006) examined the patients’ awareness and satisfaction with the information provided by clinicians*. *The study found that the majority of patients (69%) were not satisfied with the *information *they received from their health care providers, while 64 % were* willing to receive information about their disease and had positive attitude about providing essential information regarding cancer and cancer care. In addition, 82% of patients believed that the most appropriate person to tell patient the truth is the treating physician*^7^*.*

*In many countries, especially in Asia, patients are not told the truth about the diagnosis of cancer, because breaking bad news, if done prematurely, can irreversibly destroy the patient’s hope. In such societies, family members make decision without the patient's input* ^5^ *. In a study of Shahidi et al. (2007), the majority of family members were of the opinion that the patients are too frail to know the truth and were opposed to telling the diagnosis to the patients* ^26^ *. In another study, family members believed that patients should be told the truth gradually during stages of therapy and based on their psychological state. According to physicians and nurses, family members are the main obstacles to revealing the truth to cancer patients* ^17^.

**The effects of truth telling on patients**

The diagnosis of cancer can be overwhelming *physically, emotionally, *or psychologically not only for patients but also for their families ^27^. A survey on cancer patients (n=20) showed that they experienced the state of shock after being informed of diagnosis^28^.Tavoli et al. (2007) performed a cross-sectional study to assess anxiety and depression in patients (n=142)with gastrointestinal cancer^29^.

Anxiety score in patient who were aware of their diagnosis was 9.1, while it was 6.3 in patients who were not aware. Higher score of depression^1^^,^^10^ was also observed in patients who were told the truth compared to those who were not told^9^^,^^10^. The study showed that patients who were informed of their diagnosis had higher levels of anxiety and depression^29^.

The study of quality of life in patients (n=129) with lung cancer, 23% were aware and 77% were unaware, showed no significant difference in social performance, pain, emotional reaction, social isolation and physical activity between the two study groups. This study also noted that sleep disturbances were more common among patients informed of their diagnosis, but, totally, no statistically significant difference was found in quality of life between the two groups^30^. A study conducted by Tarighat et al., (2006) showed no statistically significant association between anxiety / depression and awareness of diagnosis although 30% of patients had anxiety and 17% had depression^7^. The outcomes of the study by Seyyed Rasouli et al. (2014) revealed that there was a discrepancy between positive perception of prognosis and incurable nature of cancer in affected patients. Meanwhile, positive perception caused them to experience a deeper spiritual level. There was a statistically positive correlation between the perception of prognosis and spiritual health among cancer patients^31^. 

Today, in many countries, patients are fully informed about their true diagnosis, but truth disclosure is not a common practice in Eastern countries^5^.It seems that truth disclosure has a different pattern in our country. Findings have shown that most of patients and nurses have positive attitudes toward truth disclosure^13^. They believe that truth telling can facilitate the process of treatment in cancer patients^32^. Recent studies have shown that although the trend toward full disclosure is increasing in many countries, especially in Asia^20^^,^^21^, many healthcare providers find it difficult to break bad news to patients or their family members. But when they were asked to tell the truth, they preferred to use indirect strategy and vague or ambiguous language^15^ to deliver bad news^20^.

The majority of physicians withhold the truth from the patients and give information to family members due to worries about negative impact of diagnosis on patients, cultural condition, lack of time and family’s wish to do so^7^^,^^11^. There is no detailed guideline on information disclosure in Iran, showing that although truth disclosure is an important basis for effective communication health care providers find it difficult to disclose the diagnosis of cancer to patients^29^. Personnel have not been trained how to tell the truth and providing curriculum with emphasis on appropriate transfer techniques along with communication skills have been offered for physicians^20^. The family members of Iranian patients believe that diagnosis disclosure will cause the patient's condition to deteriorate^26^ so, they are opposed to telling diagnosis to the patients and try to make decisions instead of patients^5^. They believe that patients should be told the truth gradually during stages of therapy and based on patient's psychological state ^16^ and they are concerned that the patients are too frail to know the truth^5^.

However, the results of studies have shown that disclosure of diagnosis has no impact on hope and quality of life in cancer patients^29^. As the patient's individual autonomy is not considered and the family is given priority in Iran, it is still common for the diagnosis of cancer to be concealed from the patients like most Asian countries. Studies show that cancer patients are dissatisfied with the amount of information they receive^7^^,^^10^ and approximately half of cancer patients are informed of their diagnosis^33^. Meanwhile, they prefer to be told the truth by the physician and seek to have information disclosed to them first^10^. It is suggested that the disclosure of cancer diagnosis be done by a physician and in the presence of the family members^23^. The studies have also revealed that informed patients may be involved in treatment decisions as well, which improves the patient’s quality of life ^10^.

Moreover, the most appropriate person to tell the patient the truth and relevant information is the physician^7^^,^^10^^,^^23^^,^^26^. Patients have the right to be fully informed about their diagnosis or prognosis and progress of treatment. Findings show that most of cancer patients, directly or indirectly, will be informed of their diagnosis and the nature of their illness^28^.

## CONCLUSION

 It is helpful to remove the emotional barriers to give the patients the information they desire to know about their diagnosis and it seems that cultural background and the way patients are informed of an initial diagnosis of cancer have a dramatic effect on the quality of life. In order to reduce the physical and emotional harm in truth disclosed patients, it is necessary to train staff, use new treatment methods and coping techniques to deal with the unpleasant side effects. Results of many recent studies have shown that caregivers are unable to tell the truth to cancer patients because of inadequate education in communication skills and patients are not provided with sufficient information about their diagnosis and process of treatment.
